# Advances in Biomimetics: Combination of Various Effects at Different Scales

**DOI:** 10.3390/biomimetics8030329

**Published:** 2023-07-24

**Authors:** Stanislav N. Gorb, Giuseppe Carbone, Thomas Speck, Andreas Taubert

**Affiliations:** 1 Department of Functional Morphology and Biomechanics, Zoological Institute, Kiel University, D-24118 Kiel, Germany; 2 Dipartimento di Meccanica-Matematica-Management DMMM, Campus, Via Orabona 4, 70125 Bari, Italy; giuseppe.carbone@poliba.it; 3 Plant Biomechanics Group, Botanic Garden, Faculty of Biology, University of Freiburg, Schänzlestraße 1, D-79104 Freiburg, Germany; thomas.speck@biologie.uni-freiburg.de; 4 Institute of Chemistry, University of Potsdam, Karl-Liebknecht-Str. 24-25, D-14476 Golm, Germany; ataubert@uni-potsdam.de

## 1. Introduction

Biomimetics (bionics, bioinspired technology) refers to research on living systems and attempts to transfer their properties to engineering applications. Biological materials, structures, and processes are predominantly based on the combination of various effects at different scales: from the nano- and microscales, through the mesoscale, and, finally, to the macroscale. This Special Issue is devoted to the latest advances in biomimetics in all its subfields: (1) materials and structures; (2) design, construction, and devices; (3) surfaces and interfaces; (4) architecture and climatization; (5) locomotion and bioinspired robotics; (6) sensorics, information processing, and control; (7) chemical biomimetics; and (8) energy biomimetics. The guest editors encouraged the submission of manuscripts that explore the relationships between these mentioned topics, and especially those devoted to the development of biomimetic methodology. This paper collection contains contributions from biological fields focusing on the proper identification of the underlying principles in nature, and manuscripts that apply the findings on existing systems to modern technologies.

This inaugural Special Issue of *Biomimetics*, celebrating its first impact factor (3.743, June 2022), contains theoretical, experimental, and review contributions from researchers from the fields of biology, physics, material science, and engineering, who are engaged in this fast-growing field of science.

## 2. Discussion of the Papers

It is important to highlight the review paper on heterogeneous nucleation in protein crystallization by Hao Liu and coworkers (2023) [Contribution 1], and the review paper on the recent progress in bioinspired antibacterial surfaces for biomedical applications by Xiao Yang et al. (2022) [Contribution 2]. Protein crystallization technology is widely used in many fields, and the key to successful crystallization is nucleation in the protein solution. The first review is focused on the key factors influencing protein nucleation, such as the precipitating agent, temperature, solution concentration, and pH. It summarizes crystallization methods and their applications in the crystallography and biopharmaceutical fields (Liu et al., 2023). The second review deals with bacterial fouling, which has become an urgent global challenge in the medical field. In nature, many organisms have evolved numerous surfaces with specific properties to prevent bacterial settlement, and this review highlights biological antibacterial surfaces (Yang et al., 2022). The recent progress in the bioinspired antibacterial strategies and biomedical applications of these antibacterial surfaces is discussed.

The adhesion and wettability of surfaces remain hot topics in biomimetics research. Three contributions in this Special Issue deal with these problems. The paper by Can Gao et al. (2023) [Contribution 3] studies the effects of wettability and adhesion on water drainage. The authors report solid surfaces with high-adhesion hydrophilic margins and hydrophobic margins that stably pin the air–water–solid triple-contact lines at the solid bottom and solid margin, respectively, and then drain water faster through stable water channels. This article provides insights into drainage system design for various applications. The paper by Carolina Casagualda et al. (2023) [Contribution 4] deals with L-DOPA, a catecholic amino acid, present in high amounts in mussel foot proteins and capable of changing the physicochemical properties of surfaces. The authors present a mussel-inspired approach to controlling the wettability of surfaces, which enables the development of coatings with various applications, such as textiles for oil-spill water treatment and multifunctional colorless coatings (Casagualda et al., 2023). Karuppiah Nagaraj et al. (2022) [Contribution 5] studied the effects of surfactant complex ions on liposome vesicles. The results differed depending on the presence of hydrophobicity and were interpreted based on the self-aggregation, hydrophobicity, and charge densities of the co-ligand and reactants with opposite charges (Nagaraj et al., 2022).

Biological tissues may show promising biomimetic strategies for the synthesis of scaffolds in tissue engineering. The work by Elisabetta Rosellini et al. (2022) [Contribution 6] aimed at the production and characterization of small biomimetic and bioactive tubular scaffolds for potential application in vascular tissue engineering. The scaffolds were synthesized using a molding technique, starting from a gelatin/gellan/elastin blend mimicking the composition of the extracellular matrix of native blood vessels. Then, the scaffolds were functionalized using different bioactive peptides and later characterized using physicochemical, mechanical, functional, and biological approaches. The developed scaffolds showed good hydrophilicity, an elastic behavior similar to that of natural vessels, no cytotoxic activity, and high suitability for sterilization (Rosellini et al., 2022).

Since biological materials and structures are always composites at several hierarchical levels, they are good examples for creating novel bioinspired stiff-to-compliant multi-materials with enhanced mechanical behavior. Dolev Frenkel et al. (2022) [Contribution 7] used a multi-material 3D printing approach to design complex interfaces that involve a combination of stiff and compliant materials. The authors found that the bioinspired interfaces significantly increased the strain, toughness, and tensile modulus compared with the simple interface. These bioinspired interfaces can be potentially used in the biomedical and robotics fields (Frenkel et al., 2022).

The bioinspired synthesis of nanoparticles remains a rapidly growing field of nanotechnology. The green synthesis and structural characterization of neodymium selenide nanoparticles are the topics of the paper by Abu A. Ansary et al. (2022) [Contribution 8]. For this purpose, they used a reducing agent extracted from the fungus *Fusarium oxysporum.* The hydrodynamic radii of biogenic polydispersed neodymium selenide nanoparticles were 57 nm, with no visible sign of agglomeration, which makes them excellent candidates for applications in labeling and bioimaging technology (Ansary et al., 2022).

Plant-inspired robotics, combining principles underlying the movements, attachment, and adaptability strategies of plants, is a rather novel field of biomimetics research. Falk J. Tauber et al. (2022) [Contribution 9] took combined inspirations from snap trapping in two plant species (*Aldrovanda vesiculosa* and *Dionaea muscipula*) and designed an artificial Venus flytrap demonstrator. The novel motion pattern of the designed technical system is characterized by autonomous motion generation via changes in environmental conditions. The system is not only faster than biological models in its closing movement but also requires less energy for the execution of this movement (Tauber et al., 2022).

Since privacy is of major concern these days, in the paper by Ameer Tamoor Khan et al. (2022) [Contribution 10], the authors proposed a variant of Beetle Antennae Search (BAS) known as Distributed Beetle Antennae Search (DBAS), to optimize multi-portfolio selection problems without violating the privacy of individual portfolios. They applied bioinspired machine learning to this problem to ensure that client privacy and database secrecy remain intact. The simulation results demonstrated that DBAS not only ensures portfolio privacy but is also efficient and robust in selecting the optimal portfolios (Khan et al., 2022).

## 3. Conclusions

Due to the success of this first Special Issue of Advances in Biomimetics, the guest editor team intends to call for a second Special Issue. This new Special Issue will accept manuscripts on the following: (1) biomimetics of materials and structures; (2) biomimetic design, construction, and devices; (3) biomimetic surfaces and interfaces; (4) bioinspired architecture and climatization; (5) locomotion and bioinspired robotics; (6) bioinspired sensorics, information processing, and control; (7) biomimetic processing, optimization, and management; (8) biomimetic processing and molecular biomimetics; (9) energy biomimetics; and (10) development of biomimetic methodology.

## List of Contributions

1. Liu, H.; Zhao, Y.; Sun, J. Heterogeneous Nucleation in Protein Crystallization. *Biomimetics* **2023**, *8*, 68. https://doi.org/10.3390/biomimetics8010068.
2. Yang, X.; Zhang, W.; Qin, X.; Cui, M.; Guo, Y.; Wang, T.; Wang, K.; Shi, Z.; Zhang, C.; Li, W.; et al. Recent Progress on Bioinspired Antibacterial Surfaces for Biomedical Application. *Biomimetics* **2022**, *7*, 88. https://doi.org/10.3390/biomimetics7030088.
3. Gao, C.; Jiang, L.; Dong, Z. Effect of Wettability and Adhesion Property of Solid Margins on Water Drainage. *Biomimetics* **2023**, *8*, 60. https://doi.org/10.3390/biomimetics8010060.
4. Casagualda, C.; Mancebo-Aracil, J.; Moreno-Villaécija, M.; López-Moral, A.; Alibés, R.; Busqué, F.; Ruiz-Molina, D. Mussel-Inspired Lego Approach for Controlling the Wettability of Surfaces with Colorless Coatings. *Biomimetics* **2023**, *8*, 3. https://doi.org/10.3390/biomimetics8010003.
5. Nagaraj, K.; Sakthinathan, S.; Chiu, T.-W.; Kamalesu, S.; Lokhandwala, S.; Parekh, N.M.; Karuppiah, C. States of Aggregation and Phase Transformation Behavior of Metallosurfactant Complexes by Hexacyanoferrate(II): Thermodynamic and Kinetic Investigation of ETR in Ionic Liquids and Liposome Vesicles. *Biomimetics* **2022**, *7*, 221. https://doi.org/10.3390/biomimetics7040221.
6. Rosellini, E.; Barbani, N.; Lazzeri, L.; Cascone, M.G. Biomimetic and Bioactive Small Diameter Tubular Scaffolds for Vascular Tissue Engineering. *Biomimetics* **2022**, *7*, 199. https://doi.org/10.3390/biomimetics7040199.
7. Frenkel, D.; Ginsbury, E.; Sharabi, M. The Mechanics of Bioinspired Stiff-to-Compliant Multi-Material 3D-Printed Interfaces. *Biomimetics* **2022**, *7*, 170. https://doi.org/10.3390/biomimetics7040170.
8. Ansary, A.A.; Syed, A.; Elgorban, A.M.; Bahkali, A.H.; Varma, R.S.; Khan, M.S. Neodymium Selenide Nanoparticles: Greener Synthesis and Structural Characterization. *Biomimetics* **2022**, *7*, 150. https://doi.org/10.3390/biomimetics7040150.
9. Tauber, F.J.; Auth, P.; Teichmann, J.; Scherag, F.D.; Speck, T. Novel Motion Sequences in Plant-Inspired Robotics: Combining Inspirations from Snap-Trapping in Two Plant Species into an Artificial Venus Flytrap Demonstrator. *Biomimetics* **2022**, *7*, 99. https://doi.org/10.3390/biomimetics7030099.
10. Khan, A.T.; Cao, X.; Liao, B.; Francis, A. Bio-inspired Machine Learning for Distributed Confidential Multi-Portfolio Selection Problem. *Biomimetics* **2022**, *7*, 124. https://doi.org/10.3390/biomimetics7030124.

